# Pediatric Stroke in a United Arab Emirates (UAE) Tertiary Care Center: A Retrospective Descriptive Study

**DOI:** 10.7759/cureus.95388

**Published:** 2025-10-25

**Authors:** Rahaf A Lazek, Waseem Fathalla

**Affiliations:** 1 Pediatric Medicine, Sheikh Shakhbout Medical City, Abu Dhabi, ARE; 2 Pediatric Neurology, Sheikh Shakhbout Medical City, Abu Dhabi, ARE

**Keywords:** hemorrhagic stroke, ischemic stroke, moyamoya disease, neonatal stroke, paediatric neuroimaging, pediatric neurology, pediatric stroke, stroke of unknown etiology, thrombectomy, united arab emirates (uae)

## Abstract

Background: Pediatric stroke is a rare, serious neurological emergency with evolving management paradigms. Data from the United Arab Emirates (UAE) remains limited.

Objective: To describe the clinical presentation, radiological findings, etiologies, and management patterns of pediatric stroke, with special focus on eligibility for specialized acute interventions such as mechanical thrombectomy.

Methods: This retrospective review analyzed pediatric patients below 16 years of age diagnosed with stroke and admitted or evaluated between January 2000 and January 2025.

Results: 19 pediatric stroke cases were included and classified based on neuroimaging findings. Most patients were male (78.9%), with a mean age at onset of 6.8 years. Arterial ischemic stroke was the most prevalent (89.5%) compared to venous and hemorrhagic strokes. Among the identified etiologies, moyamoya disease (47.4%) is the most common etiology, followed by cardiac disorders (26.3%). Clinical presentation varied by age group. Motor weakness (69.2%) was the most common initial manifestation in childhood and adolescent groups, whereas focal seizures were most common (80%) in neonatal stroke. Brain magnetic resonance imaging (MRI) was the primary imaging modality. Middle cerebral artery (MCA) territory involvement (76.5%) and left-sided strokes (58.8%) were most frequent. Multifocal infarcts and hemorrhagic transformations were observed in 29.4% and 17.6%, respectively. No patient received t-PA; despite the availability of mechanical thrombectomy (MT), only one patient underwent the procedure, although five patients (26%) met eligibility criteria for MT.

Conclusion: While imaging and clinical features aligned with global trends, advanced intervention utilization remained limited. These findings highlight the need for earlier recognition, standardized diagnostic protocols, and improved access to specialized stroke therapies to enhance timely management in pediatric cases.

## Introduction

Pediatric stroke is a clinical syndrome characterized by an acute onset of neurological dysfunction resulting from focal brain infarction or hemorrhage. This condition is recognized by the American Heart Association (AHA), the American Stroke Association (ASA), and the National Institutes of Health (NIH) Common Data Elements for pediatric stroke research [1,2].

Pediatric stroke is classified by age, etiology, and presentation. Perinatal stroke occurs between 28 weeks of gestation and 28 days after birth, while childhood stroke occurs from 28 days to 18 years of age. Perinatal stroke is further divided based on its timing of presentation into acute perinatal stroke, which occurs around birth, and presumed perinatal stroke, recognized later in infancy [3-5].

Pediatric stroke is relatively rare, with higher incidence rates noted during the perinatal period (5-13 per 100,000 live births) [6]. Annual incidence rates are estimated at 0.60 per 100,000 in infants under 1 year of age, 0.38 per 100,000 in children under 5, 0.32 per 100,000 in those aged 5-12 years, and 0.48 per 100,000 in adolescents [7]. Stroke is more frequently observed in boys compared to girls and has a higher incidence in Black children than in other races [8]. In a study from Saudi Arabia, the pediatric stroke rate was reported at 27.1/100,000 (children aged 1 month-12 years), whereas a study from Jordan [9,10] reported an incidence ranging from 2-3/100,000 in children younger than 5 years to 8-13/100,000 in those aged 5-14. To date, no epidemiological data have been published on stroke incidence in the UAE.

According to the International Pediatric Stroke Study (IPSS), risk factors for arterial ischemic stroke (AIS) fall into eight major categories: arteriopathy, cardiac disorders, chronic systemic conditions (CSCs), infections, acute head and neck disorders (AHNDs), acute systemic conditions (ASCs), prothrombotic states (PTSs), and chronic head and neck disorders (CHNDs). Overall, 52% of children had multiple coexisting risk factors, while 9% had no identifiable risk factors [11].

In the Vascular Effects of Infection in Pediatric Stroke (VIPS) study, the recurrence rate of arterial ischemic stroke was 6.8% at one month and 12% at one year, despite antithrombotic treatment. Arteriopathy was identified as the most significant predictor of recurrence, with moyamoya disease carrying the highest risk among all arteriopathy subtypes [12].

The clinical presentation of pediatric stroke differs from that in adults and is influenced by age and underlying cause. In neonates, stroke often manifests as seizures in the first few days of life or with nonspecific signs like lethargy and poor feeding [13]. In older children, common presentations include hemiparesis, speech or visual disturbances, altered level of consciousness, and headache [11]. Developmental age represents a unique challenge in recognizing symptoms and signs of stroke in children and requires a heightened index of suspicion.

Managing pediatric stroke remains a major challenge because most treatment approaches are derived from adult guidelines. So far, no ideal, evidence-based management strategy has been proven effective for children. However, there is an increasing trend of treating pediatric stroke with adult-approved therapies, including tissue plasminogen activator (tPA) and mechanical thrombectomy (MT) in specialized centers. Studies have shown similar safety profiles comparable to those of adult patients, providing a promising pathway for care for children with acute ischemic stroke [14]. In particular, the expanded therapeutic window for MT up to 24 hours provides an opportunity for a good proportion of children with acute ischemic stroke. Timely diagnosis and exclusion of differential diagnoses become ever more important. Increasing awareness of pediatric stroke among families and first responders is crucial, along with applying appropriate assessment scales and radiological scores that inform timely management decisions.

This study aims to review the clinical and radiological profiles of pediatric stroke cases at Sheikh Shakhbout Medical City (SSMC) in Abu Dhabi, UAE, to provide insight into the patterns of presentation, recognition, clinical care pathways, and potential candidacy for specialized treatments such as tissue plasminogen activator (tPA) or mechanical thrombectomy (MT).

## Materials and methods

This was a single-center, retrospective cohort study conducted on pediatric patients diagnosed with stroke who were either evaluated or admitted at Sheikh Shakhbout Medical City (SSMC), a tertiary care referral hospital in Abu Dhabi, United Arab Emirates, over a 25-year period from January 2000 to January 2025.

A total of 21 patients were identified through hospital records using International Classification of Diseases (ICD) codes. 19 patients met the inclusion criteria and were included in the final analysis. Inclusion criteria were pediatric patients (age < 16 years) with a confirmed diagnosis of stroke based on clinical presentation and neuroimaging. Exclusion criteria were patients with alternative diagnoses or stroke mimics. Two patients were excluded, one with traumatic axonal injury following a road traffic accident (RTA) and the other with a transient ischemic attack.

Data were extracted from electronic medical records and included demographics (age at stroke onset, current age, sex, and nationality), clinical presentation, neurological signs at admission or evaluation, stroke location, underlying etiologies, risk factors, diagnostic imaging (MRI, magnetic resonance angiography [MRA], magnetic resonance venography [MRV], computed tomography [CT]), treatments, and outcomes. Laboratory investigations such as hematology, biochemistry, immunology, genetics, and infectious workups were performed as clinically indicated.

Data were analyzed descriptively to identify demographic patterns, age-specific symptoms, and underlying etiologies. Clinical presentations were categorized by age groups to identify the most common symptoms per age, and the underlying etiology into major categories for comparative analysis. We relied on neuroimaging to identify stroke topography and distinguish between patients with acute versus chronic events. Complications such as stroke recurrence or sequelae were tracked over time.

## Results

A total of 19 pediatric patients diagnosed with stroke were included in this study. The majority, 11/19 (57.9%), were Emiratis. Most were male, 15/19 (78.9%). The mean age at stroke onset was 6.8 years, with an age range from 20 hours to 15 years (Table 1).

**Table 1:** Demographic characteristics of the study cohort. Data are presented as mean (range) or frequency (percentage).

Variable	n (%) / Mean (Range)
Age at onset (years)	Mean 6.8 (range: 20 hours to 15 years)
Sex: Male	15 (78.9%)
Sex: Female	4 (21.1%)
Nationality: Emirati	11 (57.9%)
Nationality: non-Emirati	8 (42.1%)

Distribution by age group

The majority of children had their stroke in childhood (1-12 years), 10/19 (52.6%), followed by perinatal stroke, 5/19 (26.3%), adolescents, 3/19 (15.8%), and infancy, 1/19 (5.3%).

Presentation by age group

In the neonatal age group, 4 out of 5 patients (80%) presented with an acute onset of focal seizure of unknown etiology, while one patient (20%) presented with vomiting and irritability.

The one patient in the infancy group was referred for evaluation at five months of age and was found to have a presumed perinatal ischemic cerebral injury. This patient had pulmonary stenosis and underwent cardiac surgery with a secondary hypoxic ischemic injury due to prolonged cardiac arrest. A subsequent MRI revealed a chronic infarct consistent with a cardioembolic stroke.

**Figure 1:**
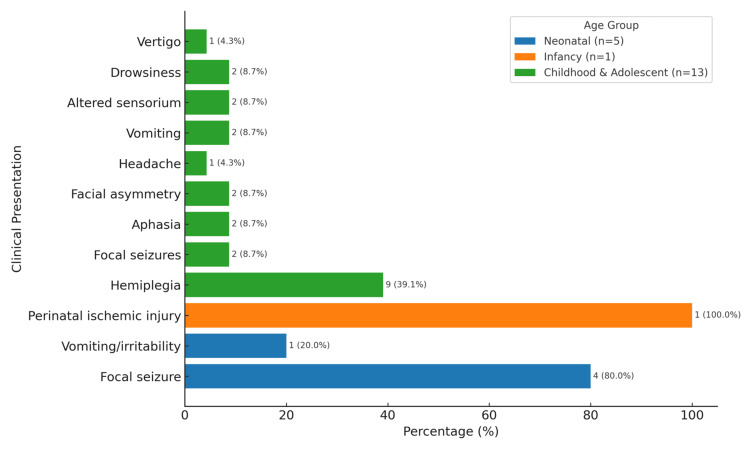
This chart illustrates the most common clinical presentation of pediatric stroke categorized by age group. Several patients exhibited more than one symptom.

In the childhood and adolescent age group (10 and 3 patients, respectively), hemiplegia was the predominant presentation with 9/13 (69.2%), followed by focal seizures (2 patients, 15.4%), aphasia (2 patients, 15.4%), facial asymmetry (2 patients, 15.4%), headache (1 patient, 7.7%), vomiting (2 patients, 15.4%), altered sensorium (2 patients, 15.4%), drowsiness (2 patients, 15.4%), and one with vertigo (7.7%). Vertigo, drowsiness, and headaches were more common in the adolescent group. Several patients exhibited more than one symptom (Figure 1).

Stroke types

A total of 17 patients (89.5%) had ischemic stroke, and two (10.5%) had hemorrhagic stroke. There was no risk factor for eight patients (42.1%), whereas the remaining 11 patients had one or more risk factors.

Etiologies

**Figure 2:**
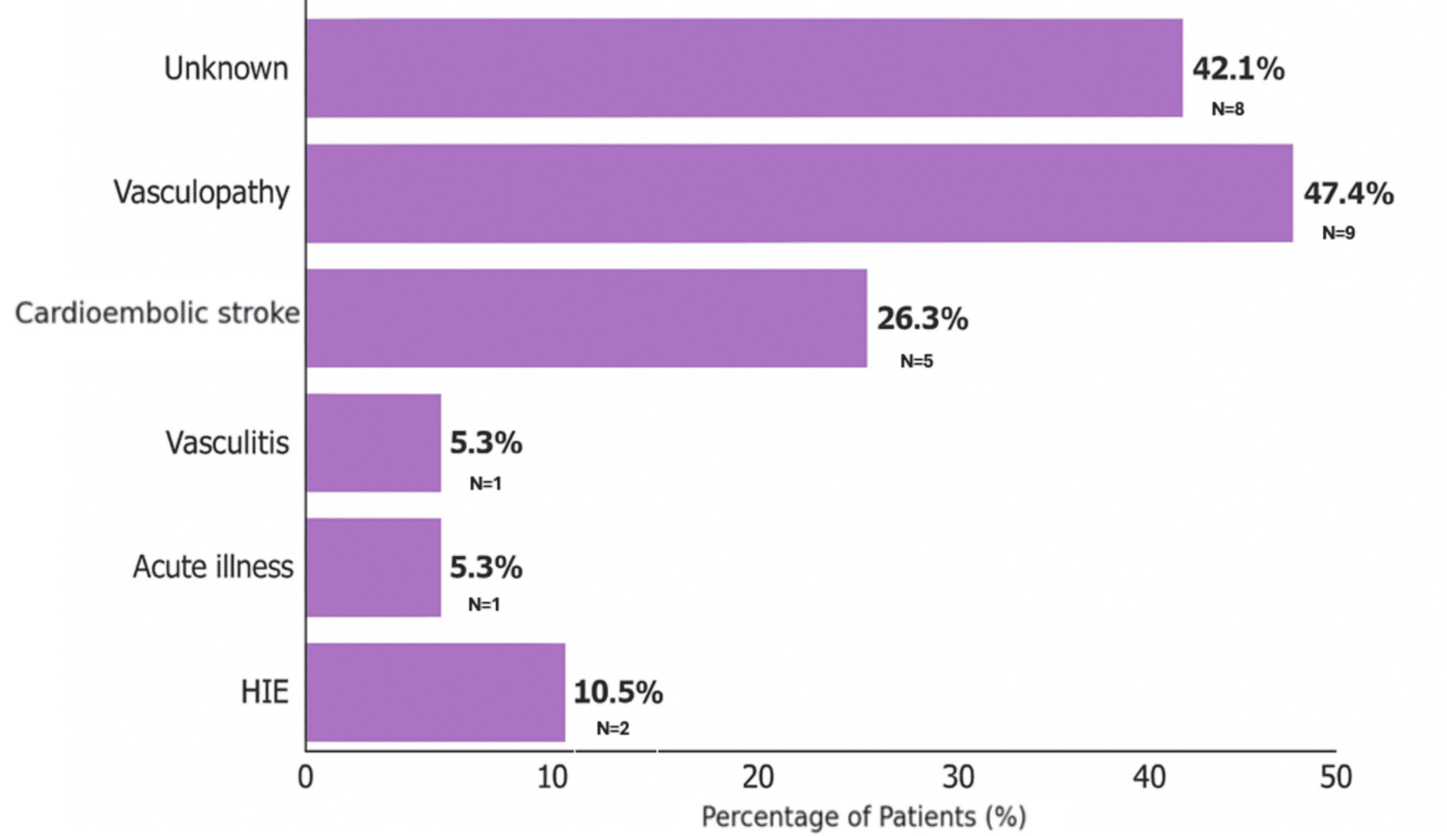
The bar graph presents the frequency of underlying causes and risk factors in 19 patients. Some patients had more than one contributing factor.

Stroke etiologies were divided into major categories based on our cohort findings (Figure 2). Vasculopathy was the most common, found in 9/19 cases (47.4%). This group included 5/19 patients (26.3%) with moyamoya disease (two of whom had associated conditions), two patients with Down syndrome, and one who had acute systemic illness. A total of 1/19 (5.3%) vasculopathy-related strokes were seen in two cases (10.5%) with Grange syndrome and Ehlers-Danlos syndrome. Cardioembolic strokes were noted in 5/19 cases (26.3%). One case (5.3%) of stroke was related to vasculitis (lupus). Hypoxic-ischemic encephalopathy (HIE) was identified in 2/19 patients (10.5%). No identifiable risk factor was found in 8/19 patients (42.1%).

Stroke subtypes and etiologies

**Figure 3:**
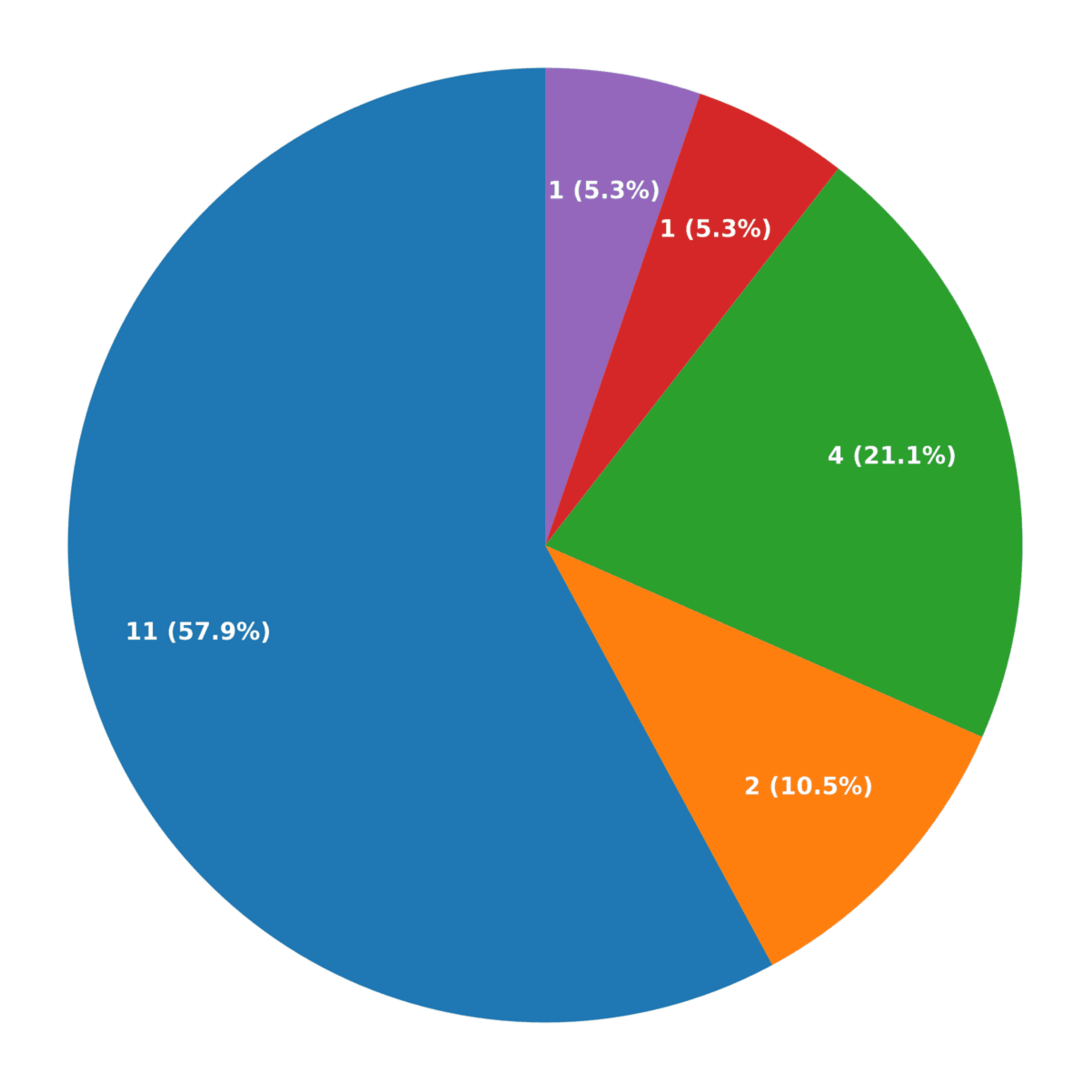
This pie chart illustrates the distribution of stroke subtypes in pediatric patients.

Arterial ischemic strokes represented the majority, seen in 17 cases (89.5%). Among the ischemic strokes, large-vessel infarcts were more common than small-vessel lacunar lesions. A total of 11 cases (57.9%) had large-vessel infarcts, whereas only two patients (10.5%) had small-vessel lacunar infarcts. Four patients (21.1%) had combined large-vessel arterial ischemic and lacunar infarcts. Intracerebral hemorrhage was less common (1 patient, 5.3%). Cerebral sinovenous thrombosis (CSVT) was diagnosed in one case as well (5.3%) (Figure 3).

Imaging findings

**Figure 4:**
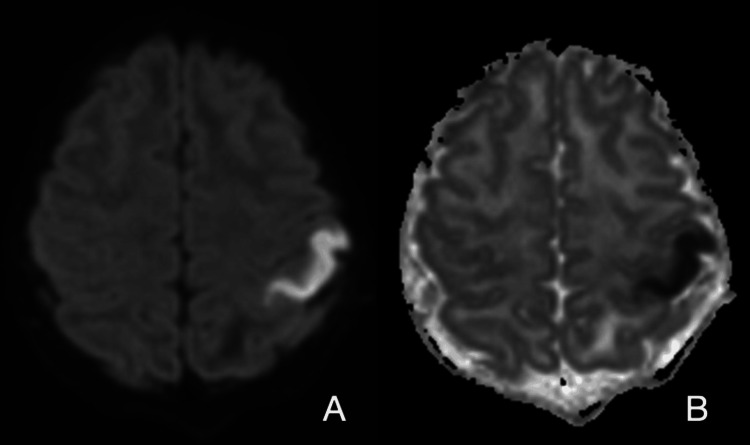
Brain MRI of acute ischemic stroke. (A) Diffusion-weighted imaging (DWI) showing restricted diffusion in the left postcentral gyrus. (B) Corresponding apparent diffusion coefficient (ADC) map demonstrating low signal, confirming acute ischemic stroke.

Magnetic resonance imaging (MRI) was the primary modality for stroke evaluation in our cohort. Computed tomography (CT) was used in eight patients, with abnormal findings reported in only half of them. MRI provided more consistent results, with diffusion-weighted imaging (DWI) and apparent diffusion coefficient (ADC) mapping clearly identifying acute ischemic changes (Figure 4). Timing of MRI relative to symptom onset differed according to the clinical setting: Among the 19 patients, MRI of the brain was performed within 12 hours (7 patients), between 12 and 24 hours (four patients), and four days after acute symptom onset (one patient). The remaining seven patients underwent MRI of the brain in the ambulatory setting as part of their workup for chronic deficits. 

Most strokes were acute, 11 (75%), while five (26%) were chronic, two subacute, and one mixed (Figure 4).

Topography

Left-sided strokes were slightly more common compared to right-sided (10 [58.8%] vs. 7 [41.2%], respectively). The middle cerebral artery (MCA) territory was the most frequently affected, observed in 13 patients (76.5%). Frontal, parietal, and temporal lobes were commonly involved, with deep structures such as the basal ganglia and thalamus affected in eight cases (47%). Watershed zone infarctions were seen in two patients, while posterior circulation involvement was seen in three patients (17.6%). One patient had findings suggestive of cerebral venous sinus thrombosis (CVST). Multifocal or bilateral lesions were observed in five patients (29.4%), and hemorrhagic transformation in three (17.6%).

Management

**Figure 5:**
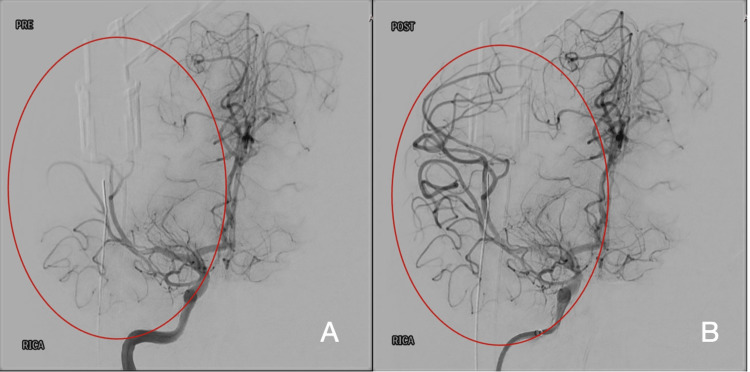
Interventional angiogram of the right middle cerebral artery (MCA) (A) Distal M2 segment of the right MCA demonstrating partial occlusion (pre-thrombectomy). (B) Markedly restored blood flow in the same segment (post-thrombectomy) following successful mechanical thrombectomy.

Most patients, 17/19 (89.5%), were managed with medical treatment, while only two (10%) underwent surgical intervention. One underwent mechanical thrombectomy (Figure 5), while the other underwent synagiosis. Among the 17 patients with medical management only, patients were treated with aspirin only (eight patients) and aspirin with a standard dose of low molecular weight heparin (four patients) immediately after excluding hemorrhagic stroke. The patients treated with aspirin continued long-term aspirin, whereas low molecular weight heparin was maintained for a range of 3-6 months. The remaining 5 of 17 with medical management only were neonates who were treated with anti-seizure medications and supportive care only. 

## Discussion

There is a lack of published research focusing on pediatric stroke in the UAE. This retrospective cohort study provides the first comprehensive analysis of the clinical, etiological, and radiological characteristics of pediatric stroke over a 25-year period at a tertiary care center in the United Arab Emirates. Overall, our findings are consistent with global data.

The majority of patients, 11/19 (57.9%), were Emirati, which is likely due to referral and access bias, as the study was conducted in a government tertiary hospital that primarily serves the local population.

Arterial ischemic strokes accounted for the majority of cases, 17/19 (89.5%), and seem to be a common presentation in patients with moyamoya disease and cardiac conditions.

In our cohort of 19 pediatric stroke cases, males were more frequently affected, 15 (78.9%), than females. This finding is consistent with previous studies, which have also reported a higher incidence of pediatric stroke in males compared to females [15].

The average age of stroke onset in our cohort was 6.8 years (82 months). This is considered higher than reported in a Saudi Arabian study, where the mean age at onset was 27.1 months [9].

Clinical presentation varied by age group. Neonates are most often presented with focal seizures, while older children are frequently presented with hemiparesis. This pattern is consistent with findings from Ranzan and Rotta, who reported focal seizures in 50% of neonates and hemiparesis in 41.3% of children with arterial ischemic stroke in their single-center study in Brazil [16].

Ischemic stroke accounted for the majority of cases in our study, 17/19 (89.5%), affecting patients with large vessel arterial ischemic lesions (LVAIL), lacunar infarcts, and mixed LVAIL and lacunar infarcts. Hemorrhagic strokes (10.5%) accounted for 2/19 cases; this aligns with findings from a study in the eastern province of Saudi Arabia, where ischemic strokes were observed in 90% and hemorrhagic strokes in 10% of 31 children [17].

For most patients who presented to the emergency department with acute neurological symptoms, stroke was not the primary consideration at the time of presentation, even when initial neuroimaging was performed. Only one patient had a clear clinical suspicion of stroke; this highlights a potential oversight in early recognition and diagnostic focus in acute care settings. Furthermore, 4/8 (50%) of initial CT scans were non-diagnostic for strokes, although CT is useful in excluding hemorrhagic stroke and other intracranial pathology. Pursuing an MRI or CT angiogram when clinically suspicious of stroke is of crucial value in order to exploit the therapeutic window for potential intervention.

In our cohort, 5 of 19 patients (26.3%) would have been eligible for endovascular thrombectomy (EVT), although pediatric NIH stroke scale (PedNIHSS) scores and time of symptom onset were unavailable. Only one patient in our cohort, 1/19 (5.3%), underwent EVT; she developed symptomatic intracranial hemorrhagic conversion. The Save ChildS Pro registry [18] included 208 children, aged 28 days to 18 years, to compare the functional outcomes of EVT versus best medical treatment. Among them, 117 (56%) patients received EVT for acute arterial ischemic stroke with a confirmed large or medium vessel occlusion. These patients had more severe strokes (median PedNIHSS = 14) and a median pre-stroke modified Rankin Scale (mRS) score of 0. Of the 117 children who underwent EVT, 104 (89%) had successful reperfusion with EVT, and 49/107 (42%) patients had complete reperfusion. Symptomatic intracranial hemorrhage affected only one patient (1%). Functional outcomes were favorable, showing a median mRS 90 days post-stroke of 1, a median PSOM at 90 days of 1, and a reduction in PedNIHSS at discharge to (-9). Additionally, persistent arterial occlusion at 24 hours occurred in only 15% of EVT patients.

Compared to eligibility based on the Save ChildS Pro study, our findings highlight a much lower EVT utilization rate, likely influenced by small sample size and by lack of clear institutional pathways of care for acute ischemic pediatric stroke. These findings underscore the importance of developing institutional protocols to support timely stroke recognition and establish clear criteria for selecting patients for endovascular thrombectomy with appropriate baseline and long-term outcome assessment tools.

Left-sided strokes and MCA territory involvement were most common, consistent with a previous study [19], which showed 36.8% left-sided strokes and 63.5% middle cerebral artery. However, we observed a higher rate of multifocal lesions (29.4% vs. 12%) and more hemorrhagic transformations. This may reflect differences in case severity or a higher prevalence of underlying etiologies that predispose to a high risk of recurrence, such as moyamoya disease/syndrome.

Our cohort’s imaging approach reflects the current guidelines recommending MRI as the primary intervention for diagnosing pediatric stroke, since the diagnostic accuracy of CT in stroke detection is suboptimal, as demonstrated in this study. This highlights the importance of having a high index of suspicion to pursue MRI imaging as a primary imaging modality or pursue it in case of a non-diagnostic CT. Consistent with our findings, a survey by Harrar et al. across pediatric stroke specialists in the U.S. and Canada also showed that most institutions prefer MRI over CT for initial evaluation [20].

This study has several limitations. First, its retrospective design makes it vulnerable to errors from incomplete documentation and reliance on medical records. Second, the small sample size and single-center setting restrict the generalizability of the results. Third, referral and healthcare access may explain the predominance of Emirati patients. Finally, standardized tools such as PedNIHSS scores and long-term outcome measures were not consistently available, constraining our ability to fully assess prognosis and treatment response.

## Conclusions

Our study reveals a similar epidemiological and clinical profile in comparison with those from regional and international studies. UAE-specific data is lacking, and a national registry for pediatric stroke may help fill this gap and inform improved practice. Ischemic stroke was the most common subtype, with vasculopathy being the main etiology, followed by cardiac disease, with a remaining significant proportion of cryptogenic strokes. Despite the availability of advanced therapies such as mechanical thrombectomy, their utilization was limited. A good percentage of our cohort would have been candidates for mechanical thrombectomy. Improved awareness, both public and healthcare system-wide; early recognition; improved diagnostic pathways; and expanded access to specialized care centers with availability of such acute interventions for pediatric stroke are strongly needed.
